# Accumulation of Co, Ni, Cu, Zn and Cd in Aboveground Organs of Chinese Winter Jujube from the Yellow River Delta, China

**DOI:** 10.3390/ijerph191610278

**Published:** 2022-08-18

**Authors:** Zaiwang Zhang, Qiong Zhang, Guoli Liu, Jian Zhao, Wenjun Xie, Shuai Shang, Jie Luo, Juanjuan Liu, Wenwen Huang, Jialiang Li, Yanpeng Zhang, Jikun Xu, Jiqiang Zhang

**Affiliations:** 1 Shandong Engineering and Technology Research Center for Ecological Fragile Belt of Yellow River Delta, School of Biological and Environmental Engineering, Binzhou University, Binzhou 256600, China; 2 School of Environmental and Municipal Engineering, Qingdao University of Technology, Qingdao 266520, China; 3 Integrated Agricultural Service Centre of Xiaobotou Town, Binzhou 256600, China; 4 Binzhou Institute of Science and Technology Innovation and Development, Binzhou 256600, China

**Keywords:** *Zizyphus jujuba* Mill. cv. Dongzao, heavy metal, pollution, health risk assessment

## Abstract

In the present study, winter jujube organs including fruit, fruiting leaf and foliage leaf, and associated soils in 14 typical orchards in Binzhou City, Shandong Province, China were collected and determined for the mass fractions of Co, Ni, Cu, Zn, and Cd. The mass fractions of Co, Ni, Cu, Zn, and Cd in plant tissues generally showed an order of Cu > Zn > Ni > Co > Cd as well as those in the soils decreased as Zn > Cu > Ni > Co > Cd. The values of single factor index and Nemerow pollution index suggested the jujube fruits were not polluted by heavy metals. Values of estimated daily intake for all the elements were far below their associated acceptable reference values, indicating no health risks would be caused by a single trace element. The results of targeted hazard quotient (THQ) of the metals in the fruits decreased as Cu > Ni > Zn > Cd accompanying total THQ (TTHQ) lower than 1 showing no hazard would be caused by those metals. Correlation analysis showed soil might not be the main source of heavy metals in winter jujube organs. Bioaccumulation factors (BAFs) for Co, Ni, Zn and Cd in fruits and leaves were far below 1 suggesting their low bioavailablities. The relatively great BAFs of Cu in the leaves might be due to the application of fertilizers and pesticides containing great amounts of Cu through soil and foliar spraying. To sum up, heavy metals tended not to be a major threat to winter jujube cultivation, and winter jujube had great edible safety.

## 1. Introduction

Chinese winter jujube (*Ziziphus jujuba* Mill. cv. Dongzao), a native fruit species in China with a long cultivation history is well known for its palatable and nutritious fresh fruit [1]. Under normal cultivation/treatment, the contents of vitamin C, soluble proteins and soluble sugar in the fresh fruit of winter jujube could be 3.05 mg/g, 0.27 mg/g and 18 mg/g, respectively, and the total acidity, sugar–acid ratio and total flavonoids could reach 0.23%, 7.8 and 0.23%, respectively, showing prominent nutritional value [2]. The fruit also contains many other nourishing substances such as cyclic adenosine monophosphate (cAMP), rutin and minerals, so it is an essential medicinal and edible fruit recommended to treat some diseases such as anemia, tumors, osteoporosis and hypertension [3,4]. Its leaf could also be used as Chinese traditional medicine. In China, winter jujube trees are mainly distributed in Shandong Province and Hebei Province [5]. Zhanhua District, geographically located in the Yellow River Delta in Shandong Province is described as the hometown of Chinese winter jujube with large cultivating areas whose fruits are of high quality [6]. In recent years, ecological protection and high-quality development of the Yellow River Basin has become a major national strategy of China making environmental protection and sustainable development an important work in the Yellow River Delta region [7].

Heavy metals, including essential elements such as Cu and Zn, and nonessential elements such as Cd, Hg and Pb, are known as typical environmental pollutants that can cause bad effects to the ecosystem and pose threats to public health if their occurrence exceeds certain doses [8]. Heavy metals cannot be degraded and thus make soil a sink for these elements [9]. Accompanying the continuous inputs of industrial fertilizers and pesticides, heavy metals have become major contaminants to the soil environment and are highly related to the quality of agricultural products due to the uptake, transport and accumulation of these elements in the crops [9,10,11]. Continuous monitoring and controlling of the heavy metals in soils and plants are essential to assure the quality and safety of fruits and vegetables [12].

Many anthropogenic processes such as fertilizer, spraying, irrigation, harvesting and storage might contribute to the metal accumulation in fruits [13]. During the cultivation of fruit trees, varieties of fertilizers such as organic fertilizer, chemical fertilizer and biological fertilizer have been widely used to improve the fruit quality [14,15]. Some fertilizers were reported/observed containing trace elements. For example, Cd might be mainly released to the orchards through P fertilizer; great amounts of Co might exist in the tire ash-based Zn fertilizer and Ni is a major component of some mineral fertilizers [14,16]. Additionally, Cu in the soils of the orchards might be due to historical pesticide application [14].

In recent years, risks caused by heavy metals through consuming fruits have resulted in rising concerns and been evaluated by researchers around the world [9,13,17,18]. Several tools such as estimated daily intake rates (EDI) and the target hazard quotients (THQ) were frequently calculated to assess potential health risks caused by consuming foods [13,17]. Previous studies have reported the residues and risks of consuming heavy metals in fruits such as apple, pear, jujube, banana, grape and so on [13,18,19]. However, little information is available for the accumulation and potential health risks of heavy metals in winter jujubes. Previous studies demonstrated that the mass fractions of heavy metals in the fruits of different jujube cultivars were below related food safety standards [20,21,22]. Investigations linking the metals in jujube organs and soil would benefit to better understanding the source, transport and fate of these traditional pollutants.

The application of fertilizers and pesticides is one of the most important management actions during the cultivation of winter jujube and may cause the new input of heavy metals in the orchard ecosystem. Additionally, those elements such as Co, Ni, Cu, Zn and Cd in the fertilizers/pesticides might be accumulated in the plant organs. The levels, accumulation characteristics and health risks of these metals should be comprehensively explored. So, in the present study, mass fractions of heavy metals in fruits and leaves of winter jujube and associated soils from several orchards in Binzhou City (Wudi County and Zhanhua District) were determined. The main objectives of this investigation were to: (1) determine the levels and distributions of typical heavy metals in above-ground organs of winter jujube; (2) evaluate the risks of heavy metals by consuming winter jujube fruits; and (3) reveal the accumulation characteristics of heavy metals in the winter jujube trees.

## 2. Materials and Methods

### 2.1. Study Areas and Sampling

**Figure 1:**
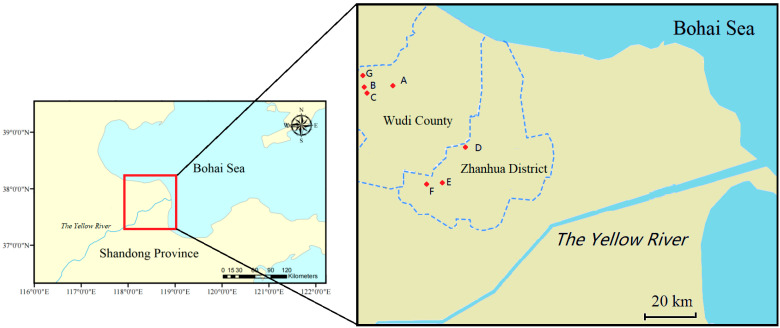
Location map of the sampling area (A: S1–S2; B: S3–S4, C: S5–S6; D: S7–S8; E: S9–S10; F: S11–S13; G: S14. A–E representing different villages).

In the present study, sampling was conducted in October 2020. Fourteen individual winter jujube orchards scattered in seven villages in Binzhou City (Wudi County and Zhanhua District), Shandong Province were chosen as sampling stations (Figure 1). At each orchard, 1 tree was randomly selected and 3 fruits (then mixed as 1 fruit sample), 6 fruiting leaves (mixed as 1 fruiting leaf sample) and 6 foliage leaves (mixed as 1 foliage leave sample) were picked. At the same time, 0–60 cm of soil around the tree was collected using a soil auger. All the samples were placed in a clean polyethylene package and stored at −20 °C until further analysis.

### 2.2. Sample Analysis

In the laboratory, the plant was firstly washed using deionized water. Then, the plant and soil samples were frozen-dried, ground and passed through a 0.5 mm sieve. Approximately 0.2000 g plant samples were transferred into the digestion vessel, adding a mixture of 6 mL HNO_3_, 2 mL HCl and 2 mL H_2_O_2_ and digested at 190 °C for 30 min using a high-performance microwave digestion system (Ethos Up, Milestone, Sorisole, Italy). Then, the evaporation was conducted using electrothermal treatment. The digested samples were diluted to a specified volume by adding 5% HNO_3_. As for the soils, approximately 0.1500 g samples were microwave-digested using a mixture of 7.5 mL HNO_3_, 2.5 mL HCl and 2 mL HF at 190 °C for 30 min as well [8]. After evaporation, the samples were also diluted by adding 5% HNO_3_. Heavy metals including Co, Ni, Cu, Zn and Cd were determined using an iCAP-RQ inductively coupled plasma mass spectrometer (ICP-MS). Mass fractions of these elements were presented in mg/kg dry weight (dw). Average water content for the jujube fruits were measured using a weight method.

Quality control measures in the metal determination processes included analysis of reagent blank, sample blank, reference materials (GBW10210, ERM-S-510204, TMRM, Changzhou, China) and duplicate samples. The recoveries of reference materials ranged from 76% to 98% (Table 1).

### 2.3. Pollution Degree Assessment

In the present study, single pollution index (*P_i_*) and Nemerow pollution index (*P_int_*) were calculated to characterize the pollution degree of a single metal and the combined pollution of the total metals in the jujube fruits, respectively. *P_i_* could be calculated using the following equation [13,23]:

*P*_*i*_ = *C_i_*/*C_si_* (1)

where *P_i_* is the pollution index of metal *i*, *C_i_* is the mass fraction of metal *i* and *C_si_* is the standard value of metal *i*. When the value of *P_i_* is greater than 1, it means the fruit sample was polluted by metal *i*; otherwise, the sample was not polluted by metal *i*.

*P_int_* could be obtained through the following equation [13]:

(2) $$P_{\mathrm{int}}=\sqrt{({P_{\max}}^{2}+Pave^{2})/2}$$

where *P_int_* is the value of Nemerow pollution index of heavy metals in a single fruit sample. *P*_max_ is the greatest *P_i_* value of metals in a single sample and *P_ave_* is the average *P_i_* values of metals in a single sample. The degree of combined metal pollution could be listed as safe (<0.7), warning (0.7–1), light (1–2), moderate (2–3) and heavy (>3).

### 2.4. Estimated Daily Intake

The possible health risk caused by those metals could be evaluated using the parameters including estimated daily intake (EDI) and target hazard quotient (THQ) [24,25]. The former was used to compare with related acceptable daily intake (ADI) suggested by the Joint Food and Agriculture Organization/World Health Organization (FAO/WHO) Expert Committee on Food Additives, while the value of the latter could demonstrate the hazard exposure of metals.

*EDI* for each element was calculated using the following equation:

*EDI* = *C_i_* × *FIR*/*B_W_* (3)

where *EDI* is the estimated daily intake (mg/kg/d); *C_i_* is the mass fraction of metal *i* in jujube fruits (mg/kg fresh weight (fw)); *FIR* is the food ingestion rate (g/d). *B_W_* is the average body weight (kg) (for adults, *B_W_* = 55.9 kg; for children, *B_W_* = 32.7 kg) [24]. The *FIR* values we selected were 8.6 g/d [13], representing annual consumption. Since winter jujube is a nutritious and delicious fruit and the children like it very much, the consumed amount of jujube was considered equal for both adults and children.

*THQ* was calculated by the following formula:

*THQ* = *C_i_* × (*E_F_E_D_FIR*)/(*B_w_A_T_RfD*) (4)

where *E_F_* is exposure frequency (365 days/year); *E_D_* is the exposure duration (20 years for adults and 5 years for children); *A_T_* is the averaging exposure time (equals 365 days/year × *E_D_*) and *RfD* is the oral reference dose (mg/kg/day). *RfD*s for Ni, Cu, Zn and Cd were 0.02, 0.04, 0.3 and 0.001mg/kg/d [26,27].

### 2.5. Bioaccumulation Factor (BAF)

To evaluate the accumulation efficiency of heavy metals in the plant organs, *BAF* was calculated. *BAF* is defined as the ratio between the metal mass fraction in the plant tissues and that in the soil [28]. The formula is

*BAF* = *C_pi_*/*C_si_* (5)

where *C_pi_* is the mass fraction of metal *i* in the jujube organs and *C_si_* is the mass fraction of metal *i* in soil.

### 2.6. Statistical Analysis

Statistical analysis was performed with SPSS 19.0 (IBM SPSS, New York, NY, USA). One-way analysis of variance (ANOVA) was used to determine the variances of metal contents among different jujube organs and the EDI and THQ of different metals in the jujube fruits. Pearson’s correlation with the two-tailed test were conducted to explore the correlation and potential sources of heavy metals in jujube organs and the soils.

## 3. Results and Discussion

### 3.1. Levels of Heavy Metals in Winter Jujube Organs and Soils

Mass fractions of heavy metals in the aboveground organs of winter jujube and associated soils are shown in Table 2. Mass fractions of Co, Ni, Cu, Zn and Pb in the jujube fruits ranged from 0.01 to 0.20, nd–2.16, 0.25–19.45, 2.62–7.82 and nd–0.084 mg/g dry weight (dw), respectively, generally showing an order of Cu > Zn > Ni > Co > Cd. Our results (converted to contents based on fresh weight according to a water content of 77%) were similar to those in winter jujube fruits in Zhanhua District reported by Gao et al. [20] and Rui et al. [21] (Table 2). To some extent, this observation might indicate that the quality of winter jujube fruit varied little over the past 10–15 years. In addition, our results were also in line with those in winter jujube fruits in some other producing regions in China such as Dagang in Tianjin City and Huanghua City in Hebei Province [29]. The above observations tended to show that winter jujube fruits accumulate relatively stable amounts of heavy metals including Co, Ni, Cu, Zn and Pb. It might also suggest that the current management measures and soil environment in the winter jujube orchards would not decrease the qualities of fruits just considering the pollution of the above metal elements. In addition, it could be observed that values for mass fractions of Cu and Cd in the fruits varied greatly with high SD. It might be due to the spatial and/or management differentiation in different orchards.

Mass fractions of Co, Ni, Zn and Cd in both the foliage and fruiting leaves were in the same order of magnitude as those in fruit. Additionally, Cu contents in the leaves were about an order of magnitude higher than those in the fruit (*p* < 0.01). The mass fractions of metals in the leaves also decreased as Cu > Zn > Ni > Co > Cd. Relatively higher contents of Cu might be due to (1) the great accumulation capacity of Cu in the leaves and (2) the application of bordeaux mixture, a cupric pesticide used against insects during a period between July and August, while the fruits become ripe in October resulting in less residues of heavy metals in the fruits.

Mass fractions of Co, Ni, Cu, Zn and Cd in the soils ranged from 19.20 to 40.38, 34.68–84.66, 53.93–96.18, 63.68–122.14 and 0.11–0.29 mg/kg, respectively, presenting an order of Zn > Cu > Ni > Co > Cd. These observations were in line with those reported in vegetable soils in Zhanjiang City [30], agricultural soils in Baiyangdian wetland [31], and soils in Huanghua City in China [32]. Mass fractions of the five metals in all the soil samples were far below their associated risk screening values for soil contamination of agricultural land in China (GB15618-2018) indicating that the pollution of these metals in the soils could be neglected. These metals in the soil would barely cause quality and safety problems for agricultural products.

### 3.2. Health and Risk Concerns

**Figure 2:**
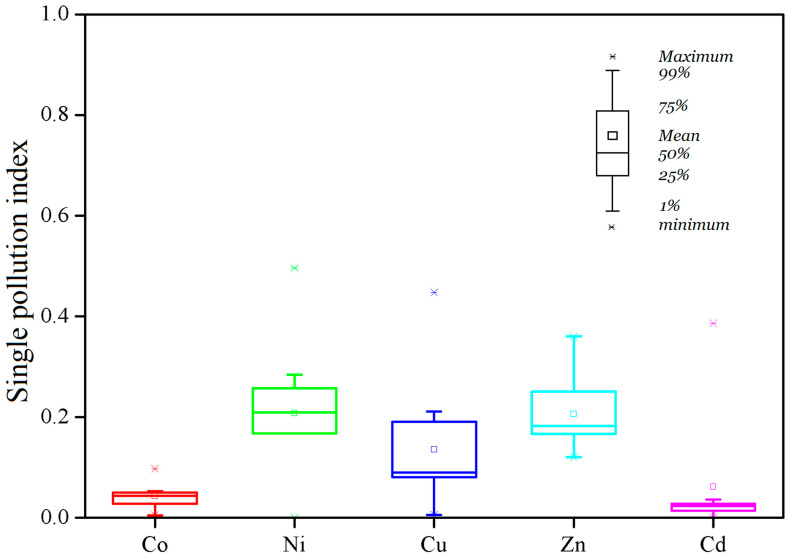
Single pollution indexes of heavy metals in winter jujube fruits.

**Figure 3:**
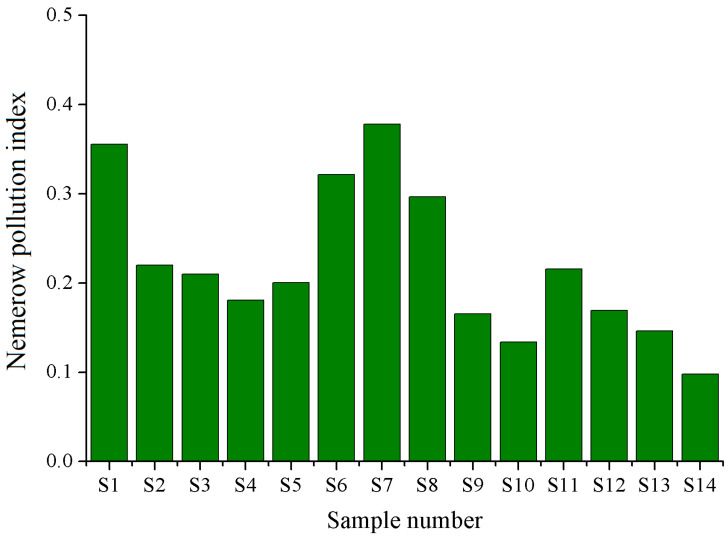
Nemerow pollution indexes of heavy metals in winter jujube fruits.

The single pollution indexes of the five elements are shown in Figure 2. The reference mass fractions of Co, Ni, Cu, Zn and Cd we selected were 0.48, 1, 10, 5 and 0.05 mg/kg, respectively [13,23,33]. It was obvious that the mass fractions of the five metals (<0.01–0.05, nd–0.50, 0.13–4.47, 0.57–1.80 and nd–0.019 mg/kg fw for Co, Ni, Cu, Zn and Cd, respectively) in all the fruit samples were lower than the related reference values. The average values of *P_i_* for Co, Ni, Cu, Zn and Cd were 0.02, 0.21, 0.14, 0.21 and 0.06, respectively, far below 1, showing that no samples were polluted by the five trace elements. It was obvious that the *P_i_* values for Co and Cd were significantly lower than those of Ni and Zn (*p* < 0.01). In addition, values of the Nemerow pollution index in the fruit samples were in the range of 0.09–0.37 with an average of 0.22 (Figure 3), among which Ni and Zn were the major contributors. Those *P_int_* values much lower than 0.7 indicated that the multiple pollution degree of heavy metals was safe. So, both the *P_i_* and *P_int_* values suggested that the jujube fruits were not polluted by heavy metals based on the food safety considerations.

In recent years, several metal elements have been removed from the list of toxic elements for food security of the World Health Organization (WHO) and most countries due to their extremely low potency to cause health problems. Cu, Zn, Co and Ni were no longer considered for their general standard in food and feed, and Cd was the only contaminant that was given a reference value in this study. So, the results of *P_i_* and *P_int_* were overestimated to a large extent, strongly suggesting the jujube fruits had a high security.

**Figure 4:**
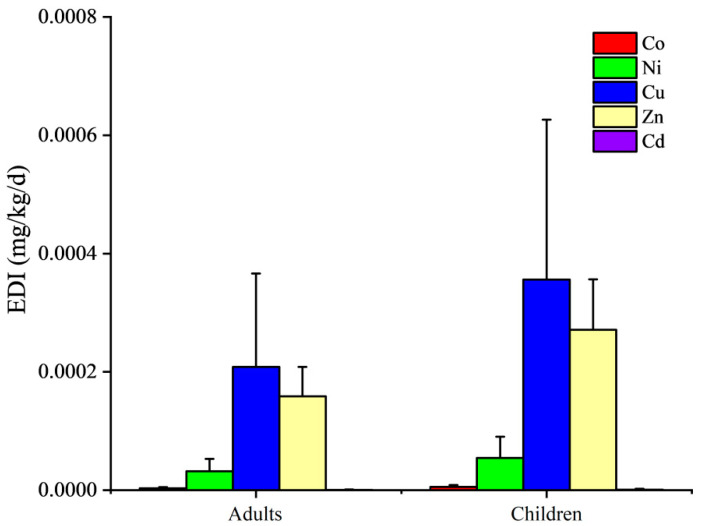
Estimated daily intake of heavy metals for adults and children.

**Figure 5:**
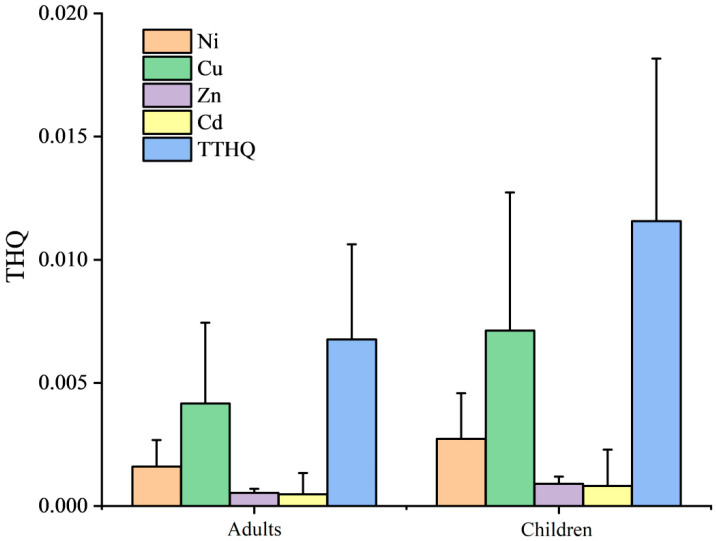
THQ of heavy metals for consuming winter jujube fruits.

The average EDI values for Co, Ni, Cu, Zn and Cd are shown in Figure 4. Acceptable daily intake (ADI) values for Ni, Cu, Zn and Cd were 0.02, 0.05–0.5, 0.3–1 and 0.001 mg/kg/d, respectively [24,33]. It was obvious that the EDI values for all the elements were far below their associated ADI values, suggesting no health risks would be caused by a single element. The THQ values of the five heavy metals followed an order of Cu > Ni > Zn > Cd (Figure 5). THQ values of Cu were significantly higher than those of the other elements (*p* < 0.01). In reality, the above five metal elements tended to barely cause any health risk to both adults and children. As mentioned above, the winter jujube is a type of seasonal fruit [6]. It was impossible for citizens to consume this fruit all year round. So, winter jujube could be perfectly edible considering the heavy metal issues.

There were still some limitations for the above risk assessment issues. Firstly, the consumed amount of jujube fruits (not only winter jujube) was based on an annual statistic which could not represent the precise ingestion rate of winter jujube. It might lead to some overestimation of the health risks of heavy metals by consuming winter jujube. On the other hand, winter jujube is a seasonal fruit and citizens could not consume it all year around. Thus, a comprehensive survey should be conducted focusing on the consumption of winter jujube for different customers.

### 3.3. Accumulation Characteristics of Heavy Metals

In the present study, correlation analysis was used to speculate the relationships between the five heavy metals in different jujube organs and their corresponding soils (Table 3). A significant correlation between Co and Ni in fruiting leaves and soil could be observed. However, the correlation coefficients of other elements in fruit, fruiting leaves and foliage leaves to soil were low, indicating that soil might not be the main source of those metals in winter jujube organs. Nowadays, a large number of nutrient elements as well as some heavy metals are released to the fruit orchard ecosystem through fertilization and pesticide spraying [14,34]. During the cultivation of winter jujube, fertilizers and pesticides are commonly used [15]. It was worth mentioning that foliar fertilization is an important pathway for the improvement of yield and quality of fruits [15,35]. Some foliar fertilizers contain trace elements such as Zn, Se and Ni [36,37]. Therefore, our findings tended to infer that the heavy metals in the above organs of the winter jujube were from both the soil and the foliar fertilizer.

**Figure 6:**
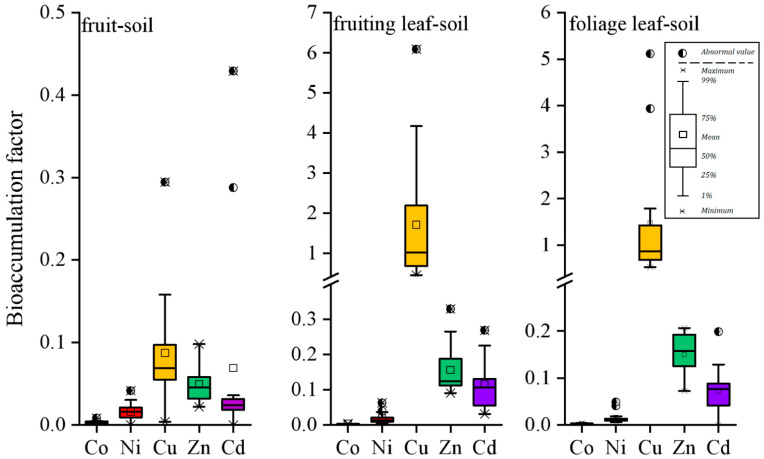
Bioaccumulation factor (BAF) of heavy metals in fruit, fruiting leaf and foliage leaf.

Figure 6 showed the BAF values of the five heavy metals in different organs of winter jujube. A general order of Cu > Zn > Cd > Ni > Co was available for all the three organs suggesting different bioavailabilities of those elements. It should be mentioned that the application of HF in the digestion process may lead to an elevation of the contents of elements in the soil samples due to the extract of trace elements in the crystal lattice of minerals, which could not be absorbed by plants in natural conditions. Therefore, the BAF values in this study might be slightly underestimated. Since silicate do not contain large quantities of heavy metals, the basic findings for BAF were still acceptable to a large extent. BAF values of the five elements in the fruit were all below 1 indicating poor enrichment of them in the fruits. Values of BAF much lower than 1 for Co, Ni, Zn and Cd were observed in the leaves. However, BAF values of Cu in the leaves were frequently observed close to 1 indicating Cu had a high enrichment capacity in the soil crop system. However, it might be a one-sided view considering Cu in leaves were mainly form soils. As mentioned above, Cu might be ascribed the application of pesticide and fertilizer that contain Cu [14]. As for the cultivation of winter jujube, bordeaux mixture, whose main chemical component is CuSO_4_ is commonly used as both of foliar fertilizer and protective fungicide [38]. Its application rate might vary among different orchards leading to a wide range of BAF values. In recent years, foliar fertilizer has gradually become a new hot spot in the study of inhibiting the absorption of heavy metals by crops because of its high solubility, good absorption and obvious effect of alleviating the absorption of heavy metals [15,35,36,37]. On the other hand, the residual Cu on the leaf surface would be easily brought into the soil by rainfall and further enriched by plants. Therefore, the present study showed that the leaves of winter jujube tended to accumulate more Cu rather than other metal elements from different pathways. It could be interesting to explore the accumulation mechanism of Cu in the winter jujube organs. So, based on some environmental protection concerns, it is of necessity to rationally use the fertilizer and pesticide involving the dose and frequency for the sustainable development of winter jujube.

## 4. Conclusions

Accompanying the wide application of fertilizers and pesticides, heavy metals have been continuously released into the farmland ecosystem. In the present study, metal elements including Co, Ni, Cu, Zn and Cd were measured in both the soil and plant samples. Our results revealed the accumulation characteristics of typical heavy metals in fruits and leaves of winter jujube from the Yellow River Delta. The mass fractions of Co, Ni, Cu, Zn and Pb in the jujube fruits were in the range of 0.01 to 0.20, nd–2.16, 0.25–19.45, 2.62–7.82 and nd–0.084 mg/g dw, respectively, showing relatively stable levels compared with previous studies. The soils proved to be safe for jujube cultivation due to the mass fractions of metal elements that were far below standard values for agricultural soil in China. The fruits were quite safe for citizens to consume based on the relatively low values for mass fractions, *P_i_*, *P_int_*, EDI and THQ of the target metal elements. The investigated metals showed low accumulation in both the fruits and leaves except for Cu. The great abundance of Cu in the leaves of winter jujube seemed to be contributed to by anthropogenic processes including the soil and foliar fertilization and pesticide spraying. It would be interesting to explore the sources and the absorption, transport and accumulation mechanisms of heavy metals in the winter jujube trees.

## Figures and Tables

**Table 1:** Information about the QC methods.

	Co	Ni	Cu	Zn	Cd
ERM-S-510204 (soil)	95.1%	78.7%	79.2%	82.8%	76.5%
GBW10210 (biota)	96.3%	81.3%	86.5%	92.2%	82.4%
RSD (duplicate samples)	5.1%	4.7%	0.5%	2.3%	1.9%

**Table 2:** Heavy metal mass fractions in jujube fruits, leaves and soils (mg/kg dw; Mean ± SD).

	Co	Ni	Cu	Zn	Cd
The present study					
Fruit (dw)	0.09 ± 0.06 ^a^	0.90 ± 0.61 ^a^	5.88 ± 4.64 ^a^	4.48 ± 1.46 ^a^	0.013 ± 0.025 ^a^
Fruiting leaf	0.08 ± 0.02 ^a^	1.21 ± 1.20 ^a^	76.16 ± 41.66 ^b^	15.10 ± 8.24 ^b^	0.021 ± 0.011 ^a^
Foliage leaf	0.07 ± 0.02 ^a^	0.91 ± 0.69 ^a^	65.25 ± 25.93 ^ab^	14.14 ± 3.17 ^b^	0.013 ± 0.010 ^a^
Fruit (fw)	0.02 ± 0.01	0.2 ± 0.14	1.35 ± 1.07	1.03 ± 0.34	0.003 ± 0.005
Soil	28.37 ± 6.51	59.49 ± 13.95	68.38 ± 11.13	96.77 ± 19.68	0.20 ± 0.05
Other studies					
Fruit (dw) [20]	-	0.4	10.1	7.75	0.027
Fruit (fw) [21]	0.01	0.03	0.23	0.79	0.001
Fruit [29]			0.66	2.37	0.002
Fruit [29]			0.62	3.08	0.004
Soil [30]		51 ± 76	34 ± 35	83 ± 69	0.19 ± 0.24
Soil [31]		36	35	90	0.21
Soil [32]		29		74	0.15
Risk screening value for agricultural soil	-	190	200	300	0.6

Note: two abnormal values of Cu in the leaves in two orchards were not included. For each element, values with same letters in one column are not significant at a *p* > 0.05 level (by Tukey test).

**Table 3:** Correlation coefficients of heavy metal elements among winter jujube organs and soils.

	Correlation Coefficient	Co	Ni	Cu	Zn	Cd
Fruit-soil	R	−0.280	−0.187	0.111	−0.306	0.016
	P	0.332	0.523	0.706	0.287	0.957
Fruiting leaf-soil	R	0.811 **	0.589 *	0.010	0.347	−0.165
	P	0.000	0.027	0.972	0.222	0.572
Foliage leaf-soil	R	−0.229	0.053	0.006	0.383	0.237
	P	0.430	0.857	0.985	0.177	0.414

Note: *n* = 14, R indicates the correlation coefficient, P indicates the significance level, * is a significant correlation at the 0.05 level (two-sided), ** is a significant correlation at the 0.01 level (two-sided).

## Data Availability

Not applicable.
